# Medical Department St. Louis University

**Published:** 1848-10

**Authors:** 


*Medical Department St. Louis University.*—The Faculty of this Institution have added two weeks to the lecture term, commencing in October next. A new chair has also been established, devoted to *Clinical Medicine and Pathological Anatomy*. W. M. McPheters, M. D., has been appointed to this chair. Dr. McPheters is one of the editors of the *St. Louis Medical and Surgical Journal.*

Prof. H. M. Bullit has retired from the school, on account of private and domestic reasons preventing him from becoming a resident of St. Louis. On the announcement of this fact to the class, a series of resolutions were adopted, expressive of their feelings of regret at his resignation, their admiration of his talents, and their thanks for his ardent exertions in their behalf. Dr. R. S. Holmes, recently of the Medical staff of the United States Army, has been appointed to the chair vacated by Dr. Bullitt.

This Institution appears to be in a flourishing condition. The Faculty state in their *annual announcement* for 1848–9, that a "new and elegant medical edifice" is in process of erection.

